# Shaping the Future of Cardiac Surgery: The Rise of Minimal-Access Techniques

**DOI:** 10.3390/jcdd12100402

**Published:** 2025-10-10

**Authors:** Heen Shamaz, Jason Ali

**Affiliations:** 1 Edinburgh Medical School, College of Medicine and Veterinary Medicine, The University of Edinburgh, Edinburgh EH16 4SB, UK; 2 Department of Cardiothoracic Surgery, Royal Papworth Hospital NHS Foundation Trust, Cambridge Biomedical Campus, Cambridge CB2 0AY, UK; jason.ali@nhs.net

Minimal access techniques are increasingly shaping the landscape of cardiac surgery. Once considered niche, these approaches have become more widespread, shifting how cardiac surgeons offer surgical options to their patients. While enthusiasm grows, so too does the responsibility to critically evaluate the evidence base, technical demands, and implications for patient outcomes.

The traditional median sternotomy remains the most commonly used approach in cardiac surgery, owing to its familiarity and safety record [1]. However, considerations re-guarding postoperative pain, recovery time, and cosmesis have driven interest in less invasive approaches [2,3], perhaps further driven by the evolution of interventional procedures such as transcatheter aortic valve implantation and mitral valve transcatheter edge-to-edge repairs. These have resulted in patients and their referrers desiring less invasive surgical alternatives to the median sternotomy.

In our special series, the breadth of cardiac surgery has been considered, and it is evident that minimal access procedures are emerging throughout the specialty. It has been understood that much of the invasiveness of cardiac surgery stems not from the incision, but from the systemic inflammatory effects of cardiopulmonary bypass (CPB) itself [4]. In this regard, when surgeons talk about minimal invasive cardiac surgery, what they often mean is minimal access. However, advances in CPB do offer the opportunity to reduce the invasiveness of cardiac surgery. Modern mini-CPB circuits aim to reduce haemodilution, inflammatory activation, and coagulation disruption. Encouraging clinical data shows reduced rates of atrial fibrillation, renal complications, and transfusion requirements. It is also noteworthy that many studies have cautioned that minimal access does not always equate to minimal invasiveness. Indeed, the technical complexity of working through reduced incisions may in fact prolong CPB or cross-clamp time, potentially offsetting the benefits of smaller incisions (Contribution 1).

Minimal access coronary revascularisation is an appealing method for treating coronary artery disease; however, the large variation in nomenclature complicates interpretation of the literature and makes comparisons with other techniques challenging. The ideal method would reap the benefits of surgical revascularisation with advantages typically seen in off-pump surgery and percutaneous coronary interventions, such as reduced pain, shorter hospital stays, faster mobilisation, and earlier return to work (Contribution 2). Minimal access techniques must not compromise the long-term patency of grafts that traditional surgical approaches reliably provide.

Valvular procedures are also seeing a shift towards minimal access techniques. Minimally invasive mitral valve surgery (MIMVS), thoracotomy or robotic techniques are becoming a popular choice in patients with low-risk degenerative valve disease (Contribution 3). Studies suggest that when appropriately selected, patients can benefit from MIMVS with durable long-term outcomes [5] (contribution 4). Similarly, minimal-access tricuspid valve surgery, whether performed via direct vision, endoscopy, or robotic methods, offers a promising solution for early intervention (Contribution 5]). In recent years we have seen the debate around open vs. transcatheter aortic valve replacement. Concerns remain regarding prosthesis durability, conduction disturbances, and stroke risk; however, as these risks are mitigated, the role of minimal-access surgical aortic valve replacement becomes increasingly attractive (Contribution 6).

The field of congenital cardiac surgery has also seen significant innovation. Techniques such as percutaneous device closure for atrial septal defects have demonstrated similar outcomes to open surgery in survival, with the added benefits of shorter hospital stays and better cosmetic outcomes (Contribution 7, Contribution 8). For younger patients, these factors are particularly relevant, and the long-term psychological impact of a less invasive approach should not be underestimated.

Robotic technologies are having a significant impact on the evolution of minimal access surgery across all surgical specialties offering precision and reduced tissue trauma. This trend towards robotic surgery is starting to gain traction in cardiac surgery. Studies have shown that while robotic techniques promise smaller incisions, faster recovery, and greater patient satisfaction, their adoption to widespread use remains limited by financial barriers, the need for specialised training, and longer operative times (Contribution 9). The evidence base is growing for these innovations; however, randomised evidence is scarce and as we know there is less room for error. Overall, the available evidence suggests that the learning curve in robotic cardiac surgery is complex and dependent on various factors, including the surgeon’s prior experience, the surgical team’s experience (consisting of cardiac anaesthesia, surgical assistants, surgical nurses, and technicians), the patient population, and the specific surgical techniques used (Contribution 10). Understanding these dynamics can inform the planning and management of surgical transitions, ensuring optimal patient care and continued improvement in surgical outcomes.

In today’s world of cardiac surgery, patient outcomes are not only related to surgical expertise. They also relate to the presence of excellent competencies in areas such as an aesthesia, intensive care, perfusion, and physical rehabilitation. Cardiac surgery is a team effort. Postoperative recovery is often cited as a key advantage of minimal access surgery, with benefits such as reduced blood loss, shorter ICU and hospital stays, improved pain management, and earlier return to baseline functional status (Contribution 11, Contribution 12). However, success depends upon careful patient selection, and a multidisciplinary learning curve is to be expected as we start integrating more of these new techniques. Alongside these challenges, Enhanced Recovery After Surgery protocols are still naïve in cardiac surgery which can cause variability in postoperative outcomes from centre to centre. In summary, minimally invasive and minimal-access techniques appear to be the future of cardiac surgery, increasingly desired by patients who perceive superiority. The articles in this Special Issue demonstrate the expanding capabilities of these techniques while also exploring the challenges in developing a balanced approach to adopting innovations. As we move forward, the goal is not merely smaller incisions, but smarter surgery which is tailored to our patients’ needs and enriched by evidence-based advancement.
